# Opportunities and Limitations of Pelvic Exenteration Surgery

**DOI:** 10.3390/cancers13246162

**Published:** 2021-12-07

**Authors:** Björn Lampe, Verónica Luengas-Würzinger, Jürgen Weitz, Stephan Roth, Friederike Rawert, Esther Schuler, Sabrina Classen-von Spee, Nando Fix, Saher Baransi, Anca Dizdar, Peter Mallmann, Klaus-Dieter Schaser, Andreas Bogner

**Affiliations:** 1 Department of Gynecology & Obstetrics, Florence Nightingale Hospital, Kreuzbergstr. 79, 40489 Düsseldorf, Germany; lampe@kaiserswerther-diakonie.de (B.L.); rawert@kaiserswerther-diakonie.de (F.R.); schuler@kaiserswerther-diakonie.de (E.S.); classen@kaiserswerther-diakonie.de (S.C.-v.S.); fixn@kaiserswerther-diakonie.de (N.F.); baransi@kaiserswerther-diakonie.de (S.B.); dizdar@kaiserswerther-diakonie.de (A.D.); 2 Department of Visceral, Thoracic and Vascular Surgery, Faculty of Medicine Carl Gustav Carus, Technische Universität Dresden, Fetscherstraße 74, 01307 Dresden, Germany; juergen.weitz@uniklinikum-dresden.de (J.W.); andreas.bogner@uniklinikum-dresden.de (A.B.); 3 Department of Urology and Pediatric Urology, Helios Faculty of Medicine Wuppertal, Universität Witten/Herdecke, Heusnerstraße 40, 42283 Wuppertal, Germany; stephan.roth@helios-gesundheit.de; 4 Department of Obstetrics and Gynecology, University of Cologne, Kerpener Str. 62, 50937 Cologne, Germany; peter.mallmann@uk-koeln.de; 5 University Center for Orthopedics, Trauma and Plastic Surgery, Faculty of Medicine Carl Gustav Carus, Technische Universität Dresden, Fetscherstraße 74, 01307 Dresden, Germany; Klaus-Dieter.Schaser@uniklinikum-dresden.de

**Keywords:** pelvic exenteration, exenteration surgery, multivisceral surgery, pelvic malignancies, advanced pelvic carcinomas

## Abstract

**Simple Summary:**

Pelvic exenteration is often the only curative treatment option for selected locally advanced tumours, and especially for recurrent cancers. Because of the heterogeneous patient population with different clinical pictures, it is not possible to standardize the indication, treatment strategy and surgical technique for this procedure. For the same reason, as well as a rather low annual number of total cases, clinical trials are notably difficult to design. However, it is important to underline the often-underestimated possibilities of surgical treatment when a R0 resection is achievable, with a low mortality and acceptable postoperative quality of life.

**Abstract:**

Purpose: The practice of exenterative surgery is sometimes controversial and has garnered a certain scepticism. Surgical studies are difficult to conduct due to insufficient data. The aim of this review is to present the current standing of pelvic exenteration from a surgical, gynaecological and urological point of view. Methods: This review is based upon a literature review (MEDLINE (PubMed), CENTRAL (Cochrane) and EMBASE (Elsevier)) of retrospective studies on exenterative surgery from 1993–2020. Using MeSH (Medical Subject Headings) search terms, 1572 publications were found. These were evaluated and screened with respect to their eligibility using algorithms and well-defined inclusion and exclusion criteria. Therefore, the guidelines for systematic reviews (PRISMA) were used. Results: A complete tumour resection (R0) often represents the only curative option for advanced pelvic carcinomas and their recurrences. A recent systematic review showed significant symptom relief in 80% of palliative patients after pelvic exenteration. Surgical limitations (distant metastases, involvement of the pelvic wall, etc.) are diminished by adequate surgical expertise and close interdisciplinary cooperation. While the mortality rate is low (2–5%), the still relatively high morbidity rate (32–84%) can be minimized by optimizing the perioperative setting. Following exenterations, roughly 79–82% of patients report satisfying results according to PROs (patient-reported outcomes). Conclusion: Due to multimodality treatment strategies combined with extended surgical expertise and patients’ preferences, pelvic exenteration can be offered nowadays with low mortality and acceptable postoperative quality of life. The possibilities of surgical treatment are often underestimated. A multi-centre database (PelvEx Collaborative) was established to collect data and experiences to optimize the research in this field.

## 1. Methods

The aim of this study is to present the current state of pelvic exenteration from a surgical, gynaecological und urological point of view.

The databases Pubmed/MEDLINE, Elsevier and the Cochrane Library were searched for studies on pelvic exenteration surgery in gynaecological, urological and colorectal cancers, published from 1993 to 2020, according to the checklists (PRISMA) from the guidelines and recommendations for meta-analyses and systematic reviews [1]. Therefore, we searched for logical combinations of MeSH search terms (e.g., “pelvic exenteration” OR “pelvic” AND “exenteration”) for the different cancer types.

1572 publications were found and the abstracts were screened for their study types and data collected. 

The inclusion criteria were an adequate statistical analysis of survival and surgical outcomes. A clear research methodology was mandatory. These criteria did not apply for those publications describing laparoscopic and robotic exenterations, as there were only a few case series available. However, the PelvEx group published a meta-analysis, which compared outcomes between open and minimally invasive surgery for pelvic exenteration based on four studies [2].

Publications for open surgery without statistical analysis and case reports were excluded, as well as studies lacking a report of the patients/surgical outcome or a clear description of the methodology.

Different variables were extracted (e.g., type of surgery: laparoscopic/robotic/open, resectional status, surgical and survival outcome, etc.).

Randomized controlled trials and studies with comparison groups were lacking for open surgery. There was only a low number of publications describing hazards or odds ratios. Hence, it was not possible to perform a meta-analysis.

## 2. Definition

Pelvic exenteration is a multivisceral procedure for locally advanced carcinomas with adjacent organ invasion in the lower pelvis. Since its first description in recurrent cervical carcinoma by Alexander Brunschwig in 1948 [3], both surgical technique and indication evolved significantly.

The surgical strategy aims for a monobloc resection of the affected organ structures (bladder, ureters, prostate, uterus, vagina, and rectum). A distinction is made between complete exenteration (with the removal of the uterus/prostate, including the bladder, vagina and the affected rectal section) and anterior (without rectal resection) or posterior exenteration (without bladder resection).

The resectability of a tumour, and thus the indication for exenteration is based on the exact anatomy, i.e., the involvement of vessels, nerves, foramina and bone structures. The surgical challenge here is to operate as radically as necessary while sparing as much tissue as possible. The indication, but also the surgical intervention, especially requires considerable expertise. Moreover, the surgical aspect and tumour entity must be considered separately, as treatment strategies may vary depending on the primary tumour and metastatic pattern. For example, in vulvar carcinomas, hematogenous metastasis is the exception, in contrast to rectal and prostate carcinoma, while the primary goal is to achieve R0 resection locoregionally, though R0 resection must generally be sought in all tumour entities.

## 3. Indications

### 3.1. Curative

For many locally advanced primary tumours of the pelvis, and especially for recurrences, pelvic exenteration remains the only curative treatment option. The most common indications are locally advanced colorectal carcinomas (CRC), cervical carcinomas and their recurrences. Left untreated, these patients often have an overall survival of only a few months after diagnosis, despite advances in chemotherapy and immunotherapy such as checkpoint inhibitors [4]. Ultimately, surgical tumour removal in sano (R0) is the only curative treatment option for this patient population [5]. Nevertheless, a standardized evidence-based indication for this procedure is not possible due to the heterogeneity of the patient population and the primary disease.

The achievability of a resection with tumour-free margins and the exclusion of distant metastases are crucial for the indication of exenteration with curative intention. To this end, a careful preoperative staging with appropriate radiologic imaging and an accurate clinical examination are essential. The imaging assessment by the surgeon can hardly be overestimated. While bladder and rectal involvement can usually be assessed adequately during physical examination (cysto- and proctoscopy), radiologic imaging is mandatory for estimating a surgical plane to the pelvic wall. The clinical examination is inferior to imaging in this regard. In addition, PET-CTs can be helpful, especially to rule out multilocular tumour manifestation or distant metastases. Burger et al. describe sensitivity values between 82% and 100% and specificity values of 91% to 100% in the detection of bladder, rectal, or pelvic wall invasion by the joint use of MRI and PET-CT [6,7]. All patients should be discussed in interdisciplinary tumour boards to ensure an optimal (neo-) adjuvant therapy.

### 3.2. Palliative Setting

Often, palliative treatment is the only choice for patients with locoregionally advanced primary tumours due to their metastatic pattern. Frequently, these patients choose surgery during the course of chemotherapy and radiotherapy because of unbearable side effects, such as uncontrollable obstructive symptoms, impending cloacal formation, pain refractory to therapy, fistulas or extensive tumour necrosis cavities with a septic situation, or recurrent bleeding with secondary anaemia.

The advantages and disadvantages of pelvic exenteration surgery must be discussed in detail with these patients and their relatives and weighed up against less invasive measures. For example, the placement of a gastric tube or PEG (percutaneous endoscopic gastrostomy) versus a colostomy, or a nephrostomy or ureteral stent placement versus a surgical urinary diversion, can be discussed here. A recent systematic review of the PelvEx Collaborative, which showed a median survival of 14 months with significant symptom relief for 80% of patients under palliative aspects, can also be considered as an argument in favour of the surgical resection of advanced tumours with adjacent organ invasion [8].

### 3.3. Exenteration as Part of a Multimodal Therapy Concept “To Gain Time”

In addition to a curative and palliative intention, oncologists increasingly balance disease chronification with a good quality of life. This applies equally to the primary as well as to the recurrent disease [9]. An example of this is shown by Khoury-Collado et al. and Lewis et al. when analysing recurrent sarcomas and endometrial carcinomas [10,11]. Lopes et al. show a two-year survival rate after exenteration of 66% for soft tissue sarcomas [12] and Schmidt et al. show a five-year survival rate of 61.4% for endometrial carcinomas [13].

### 3.4. Outlook: Laparoscopic/Robotically Assisted Exenterations

Minimally invasive surgery techniques can be chosen for certain selected patients with favourable tumour characteristics and anatomy in specialized surgical centres [2].

Table 1 and Table 2 show a selection of publications dealing with minimally invasive surgery for pelvic exenteration; most of the studies have only a small number of patients, as the technique is on the rise. Nonetheless, the good perioperative outcome is noticeable.

The available literature lacks precise data on disease-free interval (DFS) or overall survival (OS) due to the low number of cases, so that the cases described cannot be further evaluated scientifically. Nevertheless, the short-term results show that minimally invasive surgery causes a lower blood loss, shortened hospital stay, a better quality of life and a faster reintegration into daily life and work [14].

Despite these advantages, there are reasons to be cautious; in the prospective randomized LACC trial laparoscopic/robotic radical hysterectomy showed higher recurrence rates and worse overall survival compared with the open procedure for early cervical cancer [15].

Analogous to the LACC trial, it is questionable if recurrence rates are higher and the overall survival is lower due to minimally invasive surgery compared to open procedure. Since exenterative surgery is only considered for tumours that have grown beyond the organ(s), anatomical structures and certain surgical margins are difficult to identify. However, this is precisely where haptic control of the surgical target area becomes elementary. Since laparoscopic and/or robotic surgery cannot (yet) achieve this haptic control, these surgical techniques are limited in this respect and laparotomy remains the current procedure of choice for exenteration surgery.

**Table 1:** Study details of laparoscopic exenterations.

Cancer Type	Author	Number of Patients (*n*)	Years	Time of Surgery (min)	Bloodloss (in mL)	Primary Therapy	Localisation	Exclusion Criteria	Complications	R0/R1/DF
Cervix 25 (R)	Lavazzo et al. [16]	25	1995–2006	270–540	370–500	NA	NA	NA	A, UL, AL, B, SI, I, IW, F, ARF, TVT, UTI, Injury of A. iliaca interna	NA
Cervix 7, Uterus 1, Vulva 4, Urethra 1, Rectum 1; 30% (P), 70% (R)	Martinez et al. [17]	14	2000–2008	339	400	74% S, 97.6% RTx, 54.5% BTx, 62% CTx	NA	Extra-pelvin diseases, paraaortic LN-metastases, involvement of the pelvic wall	45% of urostomy and 27.9% of intestinal reconstruction	11 R0
Cervix 3 (1 × P, 2 × R), Vagina 1 (R), Urethra 1 (P)	Ferron et al. [18]	5	2000–2005	270–540	<500	2 BTx + S, 1 CTx + BTx	Central	Poor general condition, tumour size >5 cm, involvement of the pelvic wall, LN-metastases, distant metastases	2 mild complications (IW)	3 DF, 2 inguinal metastases, 1 patient died after 8 months
Cervix 1 (R)	Pomel et al. [19]	1	2003	360	200	1 RTx + CTx	Central	LN-metastases, distant metastases	NA	1 R0
Cervix 1 (R)	Pomel et al. [20]	1	2003	540	250	1 RTx + CTx	Central	LN-metastases	None	NA
Rectum 6 (4 × R, 2 × P), others 3 (gyneco-/urologic)	Uehara et al. [21]	9	2006–2014	935	830	3 none, 6 CTx	Central	History of multiple LPT, Required higher/middle amputation of the sacrum	Minor: 66.7%, major: 0% (NA)	77.8% R0
2 × Bladder, 1 × Prostata sarcoma, 8 × colo-rectal (P)	Yang et al. [22]	11	2011–2015	565	547	None	Central	Distant metastases	1 × I, 1 × DVT, 2 × UTI	9 DF, 2 patients died in the follow up time (embolism, recurrence)
23 × colo-rectal (P)	Kumar et al. [23]	15 × LSK, 8 × RA	2013–2018	640	900	None	Central	Only adenocarcinoma included, recurrent tumors, bone involvement, bilateral sciatic nerve involvement, involvement of the pelvic wall	AL, I, IW, stoma/conduit complications; 3 × revision surgery	87% R0

(R): recurrence, (P): primary therapy, (LSK): laparoscopy, (LPT): laparotomy, (min) minutes, (DF): disease free, (LN): lymph nodes, (LNE): lymphonodectomy, (AL): anastomotic leaks, (UL): ureter leak, (B): bleeding, (IW): infected wound, (F): fistula, (I): Ileus, (SI): sublieus, (ARF): acute renal failure, (DVT): deep vein thrombosis, (A): abscess, (UTI): urinary tract infection, (RTx): radiation, (CTx): chemotherapy, (BTx): brachytherapy, (S): surgery.

**Table 2:** Study details of robotically assisted exenterations.

Cancer Type	Author	Number of Patients (*n*)	Years	Time of Surgery (min)	Bloodloss (in mL)	Primary Therapy	Localisation	Exclusion Criteria	Complications	R0/R1/DF
Bladder 12 (P)	Kaufman et al. [24]	12	2004–2008	384 for RA-PE + 282 for urostomy	275	None	NA	RTx, history of extensive S	IW, SI, UTI (sepsis), constriction of the ureter, prolapse, anaemia, hypercalcaemia, rash, fever	1 × R1
Cervix 3 (R)	Lambaudie et al. [25]	3	2010	480–600	200–500	1 RTx + CTx + BTx, 2 S + RTx	Central	Extra-pelvin metastases	Perineal A, F, pyelonephritis, constriction of the ureter	3 R0
Cervix 2 (R)	Davis et al. [26]	2	2010	540	550	2 RTx	Central	Distant metastases, hydronephrosis	NA	NA
Cervix 1 (R)	Lim et al. [27]	1	2009	255 for RA-PE + 120 for Ileum conduit	375	1 RTx + CTx + BTx	Central	NA	None	NA
Cervix 14 (P, R)	Jain et al. [28]	14	2013–2019	305	135	13 × RTx + CTx 1 × none	Central	Extra-pelvin metastases, paraaortic LN-metastases, immobile tumours fixed to pelvic walls	UL, I, urosepsis, ureteric stricture, bowel perforation	14 R0, after 17.5 months 5 patients showed recurrence disease and 5 patients died
Prostata 2 (R), Rectum 1 (R)	Winters et al. [29]	3	2008–2014	570–660	350–800	1 × S, 1 × S + CTx, 1x RTx	Central	NA	Pelvic A, pyelonephrits	1 R1, 2 R0
Rectum 5, Rectum Prostata 2, Prostata 1, (P)	Smith et al. [30]	8	2016–2018	498	NA (minimal)	6 × CTx, 1 RTx, 1 × BTx	Central	NA	none	8 R0
Cervix 1 (R)	Yang et al. [31]	1	2016	700	300	S + RTx + CTx	Central	NA	none	1 R0
Cervix 6 (R)	NguyenXuan et al. [32]	6	2015–2016	402	NA	1x RTx + CTx, 1 × S + BTx, 4 × S + RTx + CTx + BTx	Central	NA	UTI, ARF, sepsis, pulmonary embolism, vaginal scar disunion, anastomosis stenosis, recto-vaginal F, 2 × revision surgery	4 R0, 1 R1, 3 recurrences during follow-up
Cervix 74 (P)	Puntam-bekar et al. [33]	74	2005–2015	180	160	None	Central	Extra-pelvin metastases, distant metastasis, involvement of the rectum, extension to the lateral pelvic wall	External iliac vein injury, internal iliac vein injury, bowel injury, intra-abdominal A, ureteral strictures, urosepsis, UL, I, IW, DVT 3 × interbowel adhesions (1 × with revision surgery)	75 R0

(R): recurrence, (P): primary therapy, (RA-PE): (robot-assisted pelvic exenteration), (LPT): laparotomy, (min) minutes, (DF): disease free, (LN): lymph nodes, (LNE): lymphonodectomy, (AL): anastomotic leaks, (UL): ureter leak, (B): bleeding, (IW): infected wound, (F): fistula, (I): Ileus, (SI): sublieus, (ARF): acute renal failure, (DVT): deep vein thrombosis, (A): abscess, (UTI): urinary tract infection, (RTx): radiation, (CTx): chemotherapy, (BTx): brachytherapy, (S): surgery.

## 4. Limitations and Risks

### 4.1. Tumour Biology Aspects

Neuroendocrine carcinomas and locally advanced malignant melanomas with primary tumour manifestation in the pelvis have an extremely poor prognosis. They usually present distant metastasis at the time of diagnosis, so that exenterative surgery should not be considered. In such instances, the patient with an unfavourable prognosis would lose quality of life due to the operation.

Parenchymatous distant metastases are generally described in the literature as a contraindication to exenteration [34,35,36,37,38,39]. However, if isolated distant metastases are present, surgical removal can be considered depending on the age and general condition of the patient. For example, sometimes single remaining liver metastases or isolated pulmonary metastases can be treated curatively after adjuvant chemotherapy [40].

**Figure 1:**
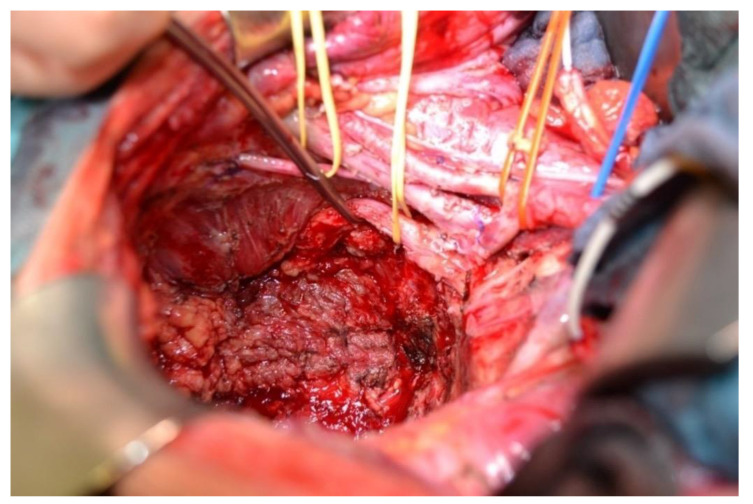
Intraoperative situs of a pelvic exenteration with resection of the ischiadic plexus with preservation of S1 and S2, as well as the obturator nerve (2 × yellow reins on the right) and placement of the internal vessels (red reins on the left) for rectal cancer recurrence (surgeon: J. Weitz).

Furthermore, involvement of the pelvic wall is no longer considered an absolute contraindication [35,38,41]. However, the concept of the pelvic wall in the context of exenteration cannot be understood as a unitary structure. From a surgical point of view, an exact definition of the infiltrating structures is necessary. From medial to lateral, the following resection levels can be distinguished: Rectum with mesorectum-vegetative nerve plexus-internal iliac artery and vein–nerve plexus/N. ischiadicus-muscle layer (M. piriformis, internal obturator muscle, M. levator) bone structures (Figure 1).

The involvement of lymph nodes, particularly in gynaecologic malignancies, is still controversial. A survey of 31 U.S. and 28 German hospitals found that lymph node metastases were more often seen as a contraindication to exenteration in the United States than in Germany [34]. Höckel et al. do not regard lymph node metastases as an absolute contraindication, though they emphasize the unfavourable prognosis when multiple paraaortic metastases are present [39].

### 4.2. Surgical Aspects

**Figure 2:**
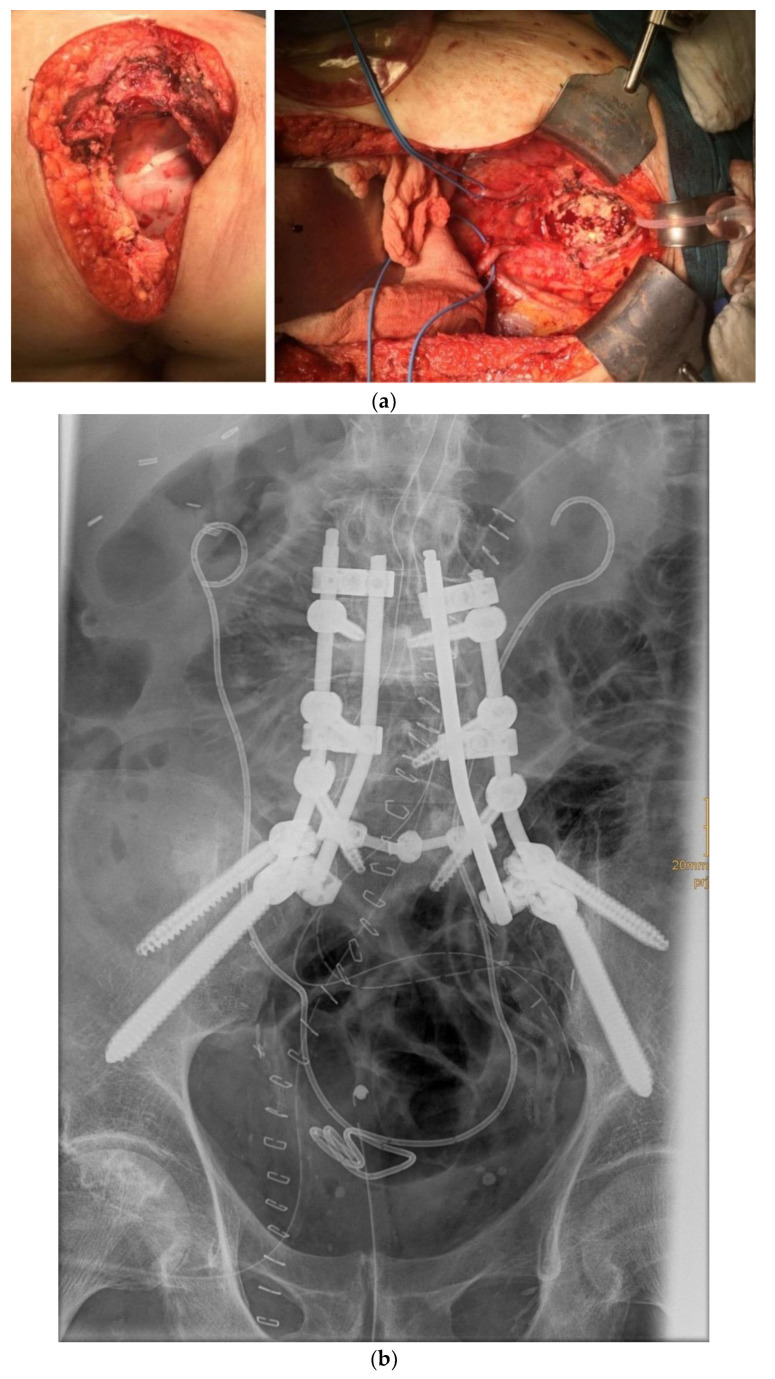
(**a**) Recurrent rectal carcinoma with infiltration of the os sacrum (surgeons: B. Lampe and D. Frank), (**b**) Recurrent rectal carcinoma with infiltration of the os sacrum; postoperative X-ray overview after stabilisation (surgeons: J. Weitz/K-D. Schaser).

The limitations of surgical possibilities are based on the absence of tumour at the resection margins (R0 vs. R1), and hence rely on the surgical experience and skills of the surgeon. Accordingly, this means that the indication should be made in qualified and experienced centres and, depending on the extent of the disease, involve an interdisciplinary team consisting of general surgery, gynaecology, urology, anaesthesia, plastic surgery, vascular surgery, trauma surgery/orthopaedics, and radiation therapy, among others. Whereas in gynaecology, the involvement of the large pelvic vessels (vasa iliaca externa and communis) and tumour-related thrombosis are considered contraindications [42], these aspects are not fundamental obstacles in general surgery (e.g., rectal cancer) [43,44]. The same can be assessed in the case of infiltration of nerval parts (nerval roots, lumbosacral plexus, etc.), which are usually considered an absolute contraindication in gynaecology [34,39,45]. Again, in general surgery, en-bloc resection, including nerval roots or the sciatic nerve, is an option for locally advanced tumours in the pelvis [46]. With the appropriate experience, a (partial) sacrectomy can also be performed in the case of infiltration of the os sacrum (Figure 2a,b). In this case, however, cooperation with an experienced orthopaedic surgeon/trauma surgeon is indispensable for stabilization, which may be necessary. Obstructive uropathy, often seen as an absolute contraindication, should be considered in context, as the obstruction may be reversible by surgery in many cases [11,39]. In particular cases, the indication for intraoperative radiotherapy, which has the potential to increase local control, must also be discussed [47].

R0/R1 resection rates show large differences within the published data, depending on the tumour type, the spreading, the state of the tumour (recurrence vs. primary disease) and the type of surgery (open vs. minimally invasive). The PelvEx group analysed data of 1293 patients with all types of tumours, showing an R0-resection rate of 71% for primary tumours and of 64% for recurrent tumours [48]. Other studies which do not differentiate between primary and recurrent diseases show variable R0 resection rates between 35,8% [49] and 81% [50] (Table 3).

### 4.3. Patient-Specific Limitations

The individual patient’s level of suffering and general condition are of paramount importance. The consequences of such a major multivisceral procedure, both for the personal body image and overall quality of life, must be discussed in detail preoperatively. The private and psychosocial environment of the patient play an important role. Psycho-oncological care should already be provided preoperatively. Quality of life can be quantified by so-called PROs (patient reported outcomes) wherein questionnaires obtain information on the state of health and the effects of interventions and treatments from the patient’s point of view. For example, an evaluation of 54 patients, 6 and 12 months following exenteration surgery for gynaecologic malignancies, showed that 82% and 79% of the patients were not only satisfied with having undergone the exenteration, but would consent to the procedure again after six and 12 months, respectively [56].

## 5. Reconstruction

**Figure 3:**
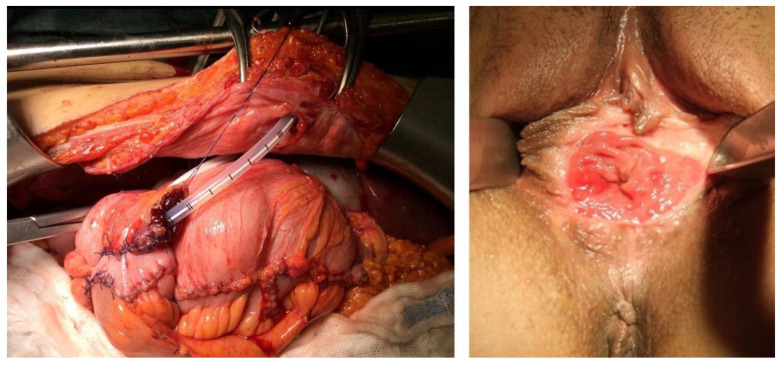
Reconstructed continent urinary reservoir by ileo-cecal segments with catheterizable outlet before anastomosis to the umbilicus (surgeons: S. Roth and B. Lampe) and formation of a neovagina—uses a section of the sigmoid colon (surgeon: B. Lampe).

The requirement for reconstruction arises from the fact that patients with a poor prognosis should not also suffer from disfigurement that reduces the quality of life. Therefore, next to free resection margins (R0-resection), the goal of exenteration surgery is to attempt the reconstruction of the bladder, rectum, and vagina, whenever possible. Numerous procedures were outlined in this regard. The spectrum ranges from, e.g., the creation of a continent urostoma with a catheterizable umbilical pouch [36,57,58,59] (Figure 3), the creation of a neovagina with a colon vaginoplasty [59] (Figure 3), the creation of a deep coloanal anastomosis with coloplasty/pouch [40,60], to the use of musculocutaneous flaps (e.g., Vertical Rectus Abdominis Myocutaneous (VRAM) flap or free flaps) to cover up defects. To prevent the problem of the so-called “empty pelvis” [61,62], omentoplasty has established itself as an excellent method, i.e., “omentum plug” [63,64].

## 6. Perioperative Morbidity and Mortality

While mortality rates in centres are 2–5% [5], the PelvEx Collaborative group describes morbidity rates ranging from 32% to 84% in an international retrospective cohort study published in 2019 [48,55]. Approximately one-third of patients experience a complication requiring intervention after exenteration [48]. These predominantly include wound-healing disorders, ileus complaints, intra-abdominal abscesses or anastomotic insufficiencies, fistulas, and ureteral stenosis with corresponding prolonged hospital stays, averaging 22.9 days [42].

### Optimization of the Perioperative Setting

A basic requirement for a reduction in perioperative morbidity and mortality is an optimal preoperative and perioperative setting. This should be achieved by specific pre-, intra-, and post-operative measures following the established ERAS (Enhanced Recovery After Surgery) concept [65].

In addition to detailed interdisciplinary support and pain management, the following measures became increasingly important in recent years:

The preoperative optimization of nutritional status based on, among other things, the measurement and correction of albumin levels, in order to minimize the increased postoperative complication rate caused by albumin deficiency [66,67].

Patient blood management (PBM) in terms of early diagnosis and therapy of anaemia, if present, to minimize blood loss and rational use of blood transfusions [68,69]. The patients’ own resources are specifically and individually conserved, strengthened, and used. The PBM should be seen as an incentive to critically evaluate and optimize local conditions. An individualized, interdisciplinary PBM bundle of measures has great potential to optimize the quality of patient care and make it safer.

In the context of intraoperative coagulation management, the ROTEM^®^ device (Werfen, Bedford, MA, USA) should be used in addition to established standard procedures. Among other things, it can detect platelet aggregation disorders, and thus distinguish between surgical bleeding and platelet dysfunction and enable targeted intervention intraoperatively [70].

The above measures were shown to improve the hospital length of stay and surgical outcomes and to shorten rehabilitation time [65]. However, challenges of complex and continuing outpatient care (stoma and wound care, mobility restrictions, etc.) also arise after hospitalization.

## 7. Conclusions

Pelvic exenteration is the only curative treatment option for selected locally advanced tumours of the pelvis and especially for tumour recurrences. However, due to the complexity of diagnosis and therapy in a highly heterogeneous patient population, no standardization is possible regarding the indication, treatment strategy and surgical technique.

In selected cases with an advanced stage of disease, distant metastases, involvement of the pelvic wall, musculature, vessels, or nerves are seldom exclusion criteria for pelvic exenteration. For patients in such a palliative setting, a pelvic exenteration can be the only choice to reach a significant symptom relief and sometimes even prolong overall survival. Yet, in general, these patients have an unfavourable prognosis.

Clinical trials are notably difficult to design and perform due to the extreme specialization and the diverse clinical pictures. As a result of the relatively low, total, annual number of cases per centre and the associated publications of minor significance, the creation of a multicentre database (PelvEx Collaborative), which makes the experiences and results comparable and advances the research in this field, has proven to be successful [54].

## Figures and Tables

**Table 3:** Literature (selected database)-R0/R1 resection rates of pelvic exenteration.

Publication	Year	Type of Cancer	No. Patients	R0/R1 Rate	Limitation/Comment
You et al. [50]	2017	Recurrent rectal cancer	229	81% R0-resection	Not only PE (>50%), but including bone and multivisceral resection
Jimenez et al. [51]	2003	Colorectal cancer	55	73% R0-resection	Only total PE
Milne et al. [52]	2013	Recurrent rectal cancer	240	74% R0-resection	Only bone/sacral resection
Kuhrt et al. [49]	2012	All types of tumours (primary and recurrent)	53	35.8% R0-resection	R1/2 higher in non-colorectal group
Bogner et al. [9]	2021	All types of tumours (primary and recurrent)	63	65.1% R0-resection	
Similis et al. (Review/meta analysis) [53]	2017	Rectal cancer (primary and recurrent)	1326	76.0% R0-resection	Studies from 1998–2014
PelvEx [54]	2018	Recurrent rectal cancer	1184	55.4% R0-resection	Data from 2004–2014, (27 centres)
PelvEx [55]	2019	Locally advanced primary rectal cancer	1291	79.9% R0-resection	Data from 2004–2014, (22 centres from 14 countries)
PelvEx [55]	2019	All types of tumour (primary and recurrent)	1293	71% R0 for locally advanced 64% for recurrent pelvic cancers	Data from 2006–2017, (22 centres)

## Data Availability

Data sharing not applicable.
